# Adrenocortical cancer: mortality, hormone secretion, proliferation and urine steroids – experience from a single centre spanning three decades

**DOI:** 10.1186/s12902-016-0095-9

**Published:** 2016-03-17

**Authors:** Jan Calissendorff, Freja Calissendorff, Henrik Falhammar

**Affiliations:** Department of Clinical Science and Education, Karolinska Institutet, Södersjukhuset, Stockholm, Sweden; Department of Molecular Medicine and Surgery, Karolinska Institutet, Stockholm, Sweden; Department of Endocrinology, Metabolism and Diabetes, Karolinska University Hospital, Stockholm, Sweden; Endocrine Section, VO Internmedicin, Södersjukhuset, Sjukhusbacken 10, 118 83 Stockholm, Sweden

**Keywords:** Adrenalectomy, Ki-67, Mortality, Survival, Adrenocortical carcinoma, Prognosis, Urinary steroid profile

## Abstract

**Background:**

Adrenocortical carcinoma (ACC) is a rare malignant disease with a poor prognosis. Our aims were to study survival and to explore prognostic markers.

**Methods:**

We retrospectively investigated the medical records of all 50 ACC patients at a single centre diagnosed between 1985 and 2012 and followed them up until 31/12/2014.

**Results:**

Of this cohort, twenty six (52 %) were females. Adrenalectomy was performed in 48 patients (96 %), and twenty seven (54 %) were treated with adjuvant cytotoxic agents. The tumor sizes ranged from 6 to 20 cm. Overall survival time was 5.5 years (0.3–19.8), the two and five-year survival was 64 and 40 %, respectively. In ENSAT stage II 25/48 patients had a median survival of 7.0 years (0.7–15.5), in stage III 8/48 this was 1.9 (0.4 – 19.8), and in stage IV 15/48 it was 1.2 (0.3–3.6) years. Seventeen patients (34 %) were still alive at the end of 2014. The total follow-up time was 8.4 (0.3–19.8) years. Cell proliferation measured with Ki-67 had a median value of 15 % (2–80) and the urinary steroid profile was clearly pathologic in 29 of 43 (67 %) tested patients. The proliferation index did not significantly predict mortality (Ki-67 ≤ 10 vs. >10 %, 9.0 vs. 3.2 years, *P* = 0.0833), but resection margins did (R1 vs. R2, *P* = 0.0066; R0 vs. R2, *P* < 0.0001). The urinary steroid profile did not predict mortality (normal vs. pathologic urine profile: median survival 6.6 vs. 3.3 years, *P* = 0.261).

**Conclusions:**

The prognosis was generally poor and macroscopically positive resection margins resulted in a worse prognosis. However, some patients were still alive many years following primary surgery with no sign of residual disease.

## Background

Adrenocortical carcinoma (ACC) is a rare malignant disease with a poor prognosis. The annual incidence has been estimated to be between 0.5 and 2 cases per million people [1]. It typically occurs during late middle age, but onset can also occur at a younger age. Symptoms can vary from abdominal pain and fatigue to hormonal symptoms if the tumor is actively secreting steroids, most commonly cortisol. ACC can also be found accidentally as an adrenal incidentaloma [2]. Computed tomography (CT) usually shows a large, often more than 5 cm, tumor in one of the adrenal glands. Treatment, which in a few cases can induce cure, is radical surgery [1]. Chemotherapy is often used. Mitotane has often been used alone or previously in combination with streptozotocin [3]. A combination with etoposide, doxyrubicin and cisplatin on top of mitotane has been proposed as first-line therapy following a large prospective randomized controlled trial [4]. Radiotherapy and targeted therapy is currently in clinical studies and has been tried in advanced disease [5]. Control of hormonal secretion is vital. Close follow-up is mandatory with repeated radiology, and in case of endocrine hyper-secretion also of blood and urine markers.

In recurrent disease repeated surgical resection of loco-regional disease is an option in selected patients [6]. The best prognostic factor after repeated surgery was time to first recurrence and radical surgery [7].

In recent publications median overall survival has in large cohorts been 35 to 38 months [8, 9], or a 5-year survival of 40 % in ENSAT stage III or 27.6 % in stage IV [10]. However, there are difficulties in deciding the malignant potential in individual tumors. Cell proliferation, gene analysis, size, hormonal activity, tumor stage and grade as well as the age of the patient and the pattern of urinary steroids are all factors which have been claimed to be of value in determining the prognosis [11–13]. Gene profiling of tumours could differentiate and identify two types of ACC with different prognosis, and the combined expression of BUB1B and PINK1 was the best predictor of overall survival [14]. The proliferative activity measured by immunostaining with the antibody MiB-1 can help to distinguish benign adrenocortical adenoma (ACA) from ACC [15]. The index is calculated by counting the percentage of positive nuclei in 1000 cells in hot spots. High proliferation in adrenocortical neoplasm can be an indicator of worse prognosis [15], especially if Ki-67 is >4 % [16] or >10 % [17]. However, long-term assessment on Ki-67 as a prognostic marker has previously only occasionally been referred to in ACC [18, 19], but Beuschlein et al. recently found Ki-67 to be the best prognostic marker for recurrence-free and overall survival in a large cohort from three European countries [17].

Urinary steroid profile (USP) may also be used in the assessment of ACC tumor activity and in the follow-up to find recurrent disease [20, 21], but no long-term investigations of this as a prognostic marker has been published. Different urinary steroids can be measured with gas chromatography–mass spectrometry [21], or by more rapid profiling of selected steroids by liquid chromatography/tandem mass spectrometry [22], although this has been questioned [23], but further studies are required. In healthy individuals urinary steroids are dominated by metabolites of androgens and cortisol. In cortisol producing adenomas the same metabolites are also seen but the levels of cortisol metabolites are increased as are the tetrahydrocortisol - tetrahydrocortisone ratios [21]. To distinguish ACC from Cushing’s syndrome, secretion from accidentally discovered incidentaloma, or congenital adrenal hyperplasia USP can be of value [24, 25].

In ACC there is a characteristic domination of precursor steroids, indicating an immature steroidogenesis. The measurement of USP can also discover steroid precursors in ACC that has been classified as hormonally inactive, based on work-up with routine serum and urine analysis. In the majority of patients with ACC raised levels of tetrahydro-11-deoxycortisol (THS), pregnanetriol and/or 3β-hydroxy-5-ene steroids are seen (which is an umbrella term for metabolites from dehydroepiandrosterone [DHA], pregn-5-ene 3β,20α-diol [pregnenolone] or pregn5-ene-3β,17α,20α-triol [17α-hydroxypregnenolone]). Androgen metabolites are also frequently elevated in ACC, demonstrated by Gröndal et al. who reported that 13/24 patients with ACC had elevated androsterone and/or etiocholanolone levels [21]. However, normal USP can be found in ACC.

Our aims were to study clinical features, survival and different markers in a cohort with ACC from a single tertiary centre to explore which factors could be used to predict the prognosis and survival. Moreover, we wanted to explore the potential benefit of measuring USP.

## Methods

All medical records of patients diagnosed with ACC from 1 January 1985 to 31 December 2012 at the Karolinska University Hospital in Stockholm, Sweden, were audited. Sixty-two patients were identified, however, 11 patients had been wrongly diagnosed and one was lost to follow-up due to emigration. Thus 50 patients remained in the follow-up analysis. All patients had ACC diagnosed based on a combination of tumour size, clinical status, endocrine evaluation, radiology, histopathology, metastases at diagnosis or at follow-up and USP. Five were not known to have ACC at the time of primary adrenalectomy. All patients were followed until 31/12 2014. Clinical outcomes, urine and blood laboratory tests, USP, imaging, histopathology, including tumour stage and proliferation index (Ki-67), and treatment were recorded. Functional status was determined by pre-operative measurements of cortisol (24 h-urine sample or serum after a 1 mg dexamethasone suppression test [DST]), aldosterone (plasma or 24 h-urine sample), catecholamine (24 h-urine sample)/metanephrines (plasma), and adrenal androgens (serum) together with clinical status. A pathologic USP was defined as an elevation more than three times the upper limit of normal compared to normal levels of androgens (androsterone, etiocholanolone) and/or cortisol metabolites and/or 3β-hydroxy-5-ene steroids. Patients with an intermediate USP were distinguished by up to three times the upper limit of the normal range for androgens and cortisol metabolites, resembling findings in adrenal Cushing’s syndrome. Survival was calculated from the date of surgery (and for those not having surgery from the date of diagnosis) to the date of death of any cause or if still alive to 31/12 2014. All data was retrieved from the electronic or microfilmed medical records, and the National Population Register was consulted to determine whether the person was still alive. Time to local recurrence was estimated from the date of first surgery to the date of the imaging revealing recurrence.

Resection margins at surgery were defined as: R0, no evidence of tumour; R1, microscopically positive; R2, macroscopically positive; RX, unknown. The ENSAT staging system was used as it is considered superior in ACC [26]. The classification defines: stage I, ACC ≤5 cm in the largest diameter and confined to the adrenal gland; stage II, ACC >5 cm without extra-adrenal invasion; stage III, presence of positive lymph nodes, infiltration to the surrounding tissue, or vascular tumour extension; stage IV, distant metastasis.

### Statistical analysis

The data analysis was made in GraphPad Prism version 6.0 (developed GraphPad Software, Inc.). Mean ± SD or median (range) was used when appropriate. All proportions were calculated discounting missing values. Survival was analyzed with the Kaplan-Meier technique and comparisons were made with the log-rank test. Patients who died without local recurrence were censored to the date of death and patients were censored to the last follow-up if local recurrence or death had not occurred. A confidence interval of 95 % was used. A *P* < 0.05 was considered significant, and when multiple comparisons were made correction according to the Bonferroni principle was performed.

## Results

Information about the background and demographic factors of the 50 patients are shown in Table 1. About half of the patients were females and about half the lesions were localized in the right adrenal. Median age at diagnosis was 59 (3–84) years. Two patients (4 %) had neurofibromatosis type 1. One patient (2 %) was a 3-year-old child with no other known disease or syndrome such as Li-Fraumeni or Beckwith-Wiedemann syndrome. He was healthy with no signs of disease at last follow-up, eight years post-surgery. Tumour size varied considerably from 6 to 20 cm with a mean size of 11.8 ± 4.4 cm. Patients were diagnosed due to localized symptoms and pain in 11 (22 %), hormonal symptoms in 19 (38 %) and/or signs of malignancy in four cases (8 %). Two cases (4 %) were discovered during surveillance for other malignancies and the remaining nine cases (18 %) had nonspecific symptoms such as pneumonia or heart failure necessitating CT. In five cases (10 %) patients had medical imaging performed for unknown reasons. Macroscopic surgical margins were R0 in 19/48 (40 %), R1 in 17/48 (35 %), R2 in 13/48 (27 %), and Rx in 1/48 (2 %). When patients were classified according to ENSAT staging 25/48 (52 %) were in stage II, 8/48 (17 %) in stage III, and 15/48 (31 %) in stage 4. In two patients ENSAT was impossible to retrieve.

**Table 1:** Background information in 50 patients with ACC

	Median	*N* (%)
Age	59 (3–84)	
Sex M/F		24/26
Hormone secreting tumor		37/45 (82 %)
Non secreting tumor		8 (18 %)
Missing hormone samples		5 (10 %)
Pathologic urine pattern		29/43 (67 %)
Right/Left		27/23
Tumor weight g	415 (27–4400)	
Size cm	11 (6–20)	
ENSAT		
Missing		2 (4 %)
Stage I		0
Stage II		25 (50 %)
Stage III		8 (16 %)
Stage IV		15 (30 %)

### Laboratory

At the time of diagnosis 11/45 (22 %) had a normal endocrine work-up (excluding USP) in urine and serum, including dexamethasone suppression test. Not all cases were tested completely, thus of assessable patients 26/37 (70 %) had signs and biochemical findings suggestive of hypercortisolism, 2/36 (6 %) findings of concomitant elevation of catecholamines (urinary noradrenaline 842 nmol/L, normal < 400, and plasma normetanephrine 1.3 nmol/L, normal <0.6, respectively), and 4/26 (15 %) with hyperaldosteronism (all had more than double the upper reference limit of plasma aldosterone and urinary aldosterone with hypokalaemia and low renin). Two patients had polycythemia with haemoglobin of 180 and 235 g/L, respectively, the former of these two had an otherwise normal endocrine work-up and the latter, a woman, had an elevated testosterone level (14 nmol/L, normal <3). In all, eight had an excess of androgens measured with testosterone and/or DHEAS, five of whom were women. Of those with androgen excess, other endocrine dysfunctions were found in five cases. In total 37/45 (82 %) of all patients had endocrine disruption and only 8/45 (18 %) had a normal biochemistry. There was missing or incomplete laboratory data in five cases (10 %).

USP displayed clearly pathological findings with increased atypical steroid metabolites in 29/43 (67 %) (stage II *n* = 13, stage III *n* = 5, stage IV *n* = 11), intermediate findings in 9/43 (21 %) (stage II *n* = 5, stage III *n* = 2, stage IV *n* = 2) and a normal pattern in 5/43 (12 %) (stage II *n* = 3, stage III *n* = 1, stage IV *n* = 1) (Table 2). In those with a normal biochemistry the USP was clearly pathologic in five cases. Thus, USP combined with traditional biochemistry resulted in 42/45 (93 %) having signs of endocrine dysfunction.

**Table 2:** Urine steroid profile in 43 patients with adrenocortical cancer

	Normal	↑	↑↑	Not reported
Androgens	10	9	19	5
3 β-hydroxy-5-enestereoids	8	6	18	9
Pregnantriol	16	7	12	8
Tetrahydro-11-deoxycortisol	16	7	12	8
Cortisol metabolites	15	10	13	5

Normal, within reference

↑ Intermediate levels ≤ three times references

↑↑ Pathologic levels > three times references

Androgens: Androsterone normal < 13 umol/24 h and/or etiocholanolone < 14.8 umol/24 h

3β-hydroxy-5-ene steroids: dehydroepiandrosterone [DHA] normal < 3.8 umol/24 h, pregnenolone < 7.4 umol/24 h or 17α-hydroxypregnenolone < 7.4 umol/24 h

Pregnanetriol < 6 umol/24 h

Tetrahydro-11-deoxycortisol (THS) < 0 umol/24 h

Cortisolmetabolites < 35 umol/24 h, these include: Tetrahydrocortison, tetrahydrocortisol, allo- tetrahydrocortisol, α-cortolon, β-cortolon, β-cortol, α-cortol

### Histopathology

Proliferation was measured with Ki-67 since 1993 (*n* = 32), and showed a mean of 18 ± 15.5 % and a median of 15 % (2–80 %). Weiss scores were not possible to analyse due to few reports.

### Treatment

Of the total, 48/50 (96 %) of the patients had adrenalectomy performed. Two patients, aged 42 and 56 years respectively, did not have adrenalectomy due to widespread metastatic disease and poor clinical status. One of these two received mitotane alone, the other mitotane in combination with etoposide, doxorubicin and cisplatin. The survival time was 9 and 18 months, respectively. At the time of primary surgery 10/48 (21 %) had known metastatic disease. These were operated on with a debulking intention. A further three cases displayed metastases at the time of surgery. Adjuvant cytotoxic agents were used within three months after surgery in 27/50 (54 %): 12 with mitotane alone (stage II: *n* = 6, stage III: *n* = 2, stage IV: *n* = 4), 10 with mitotane and streptozotocin (stage II: *n* = 5; stage III: *n* = 1; stage IV: *n* = 5), and five with mitotane, etoposide, doxorubicin and cisplatin (stage II: *n* = 2; stage IV: *n* = 3). During follow-up an additional nine patients with disseminated disease received chemotherapy, three with streptozotocin, three with streptozotocin and mitotane, and three with the combination of mitotane, etoposide, doxorubicin and cisplatin. Eight had repeated surgery due to metastatic disease.

### Survival

**Fig. 1:**
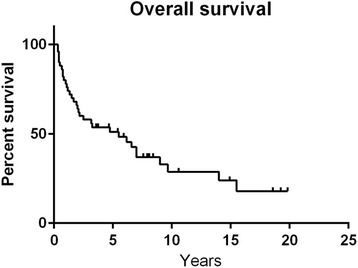
Overall survival in 50 patients with ACC

**Fig. 2:**
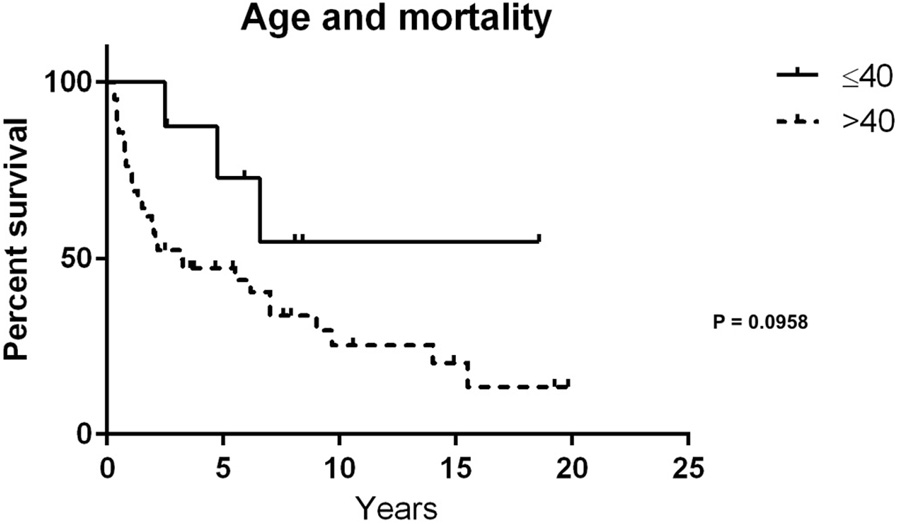
Survival stratified above (*n* = 42) or below 40 (*n* = 8) years of age at diagnosis in patients with ACC

**Fig. 3:**
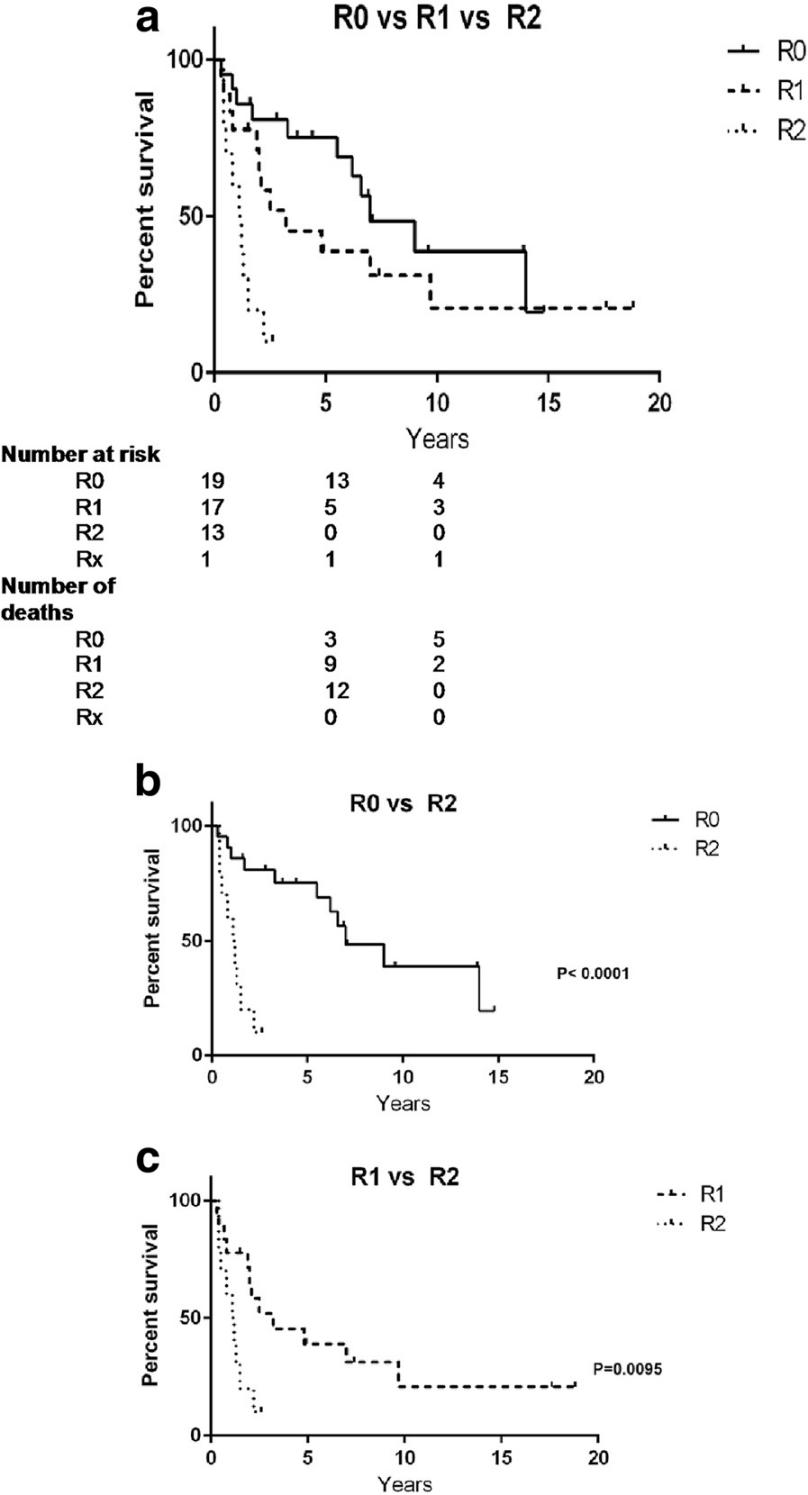
Survival according to resection margins at primary surgery in 50 patients with ACC. Two in R2 had no surgery. **a** Resection margins and survival in R0 vs R1 and R2. **b** Resection margins and survival in R0 vs. R2. **c** Resection margins and survival in R1 vs. R2

The median survival time from date of diagnosis was 5.5 years (0.3–19.8) with a two- and five-year survival time of 64 and 40 %, respectively. Overall survival is described in (Fig. 1). Survival was not significantly worse in those above 40 years old (Fig. 2). The median survival time was in ENSAT stage: II 7.0 years (0.7–15.5); III 1.9 (0.4–19.8); and IV 1.2 years (0.3–3.6). Survival between stage III vs. IV was statistically different (*P* = 0.0042). In R0 median survival was 9.0 years (0.3–14.8), in R1 4.8 years (0.3–18.8) and in R2 1.2 years (0.3–3.6). Survival between R0 vs. R2 was statistically different (*P* < 0.0001), and between R1 vs. R2 (*P* = 0.0066) (Fig. 3).

**Fig. 4:**
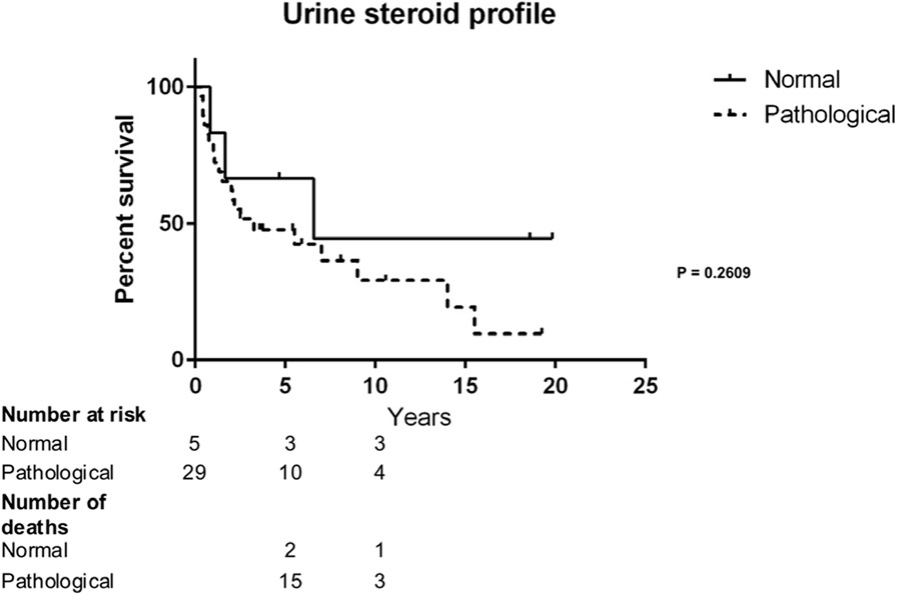
Survival in patients with ACC and normal (*n* = 5) vs. pathologic (*n* = 29) urine steroid pattern. Intermediate findings in nine patients, data not shown

**Fig. 5:**
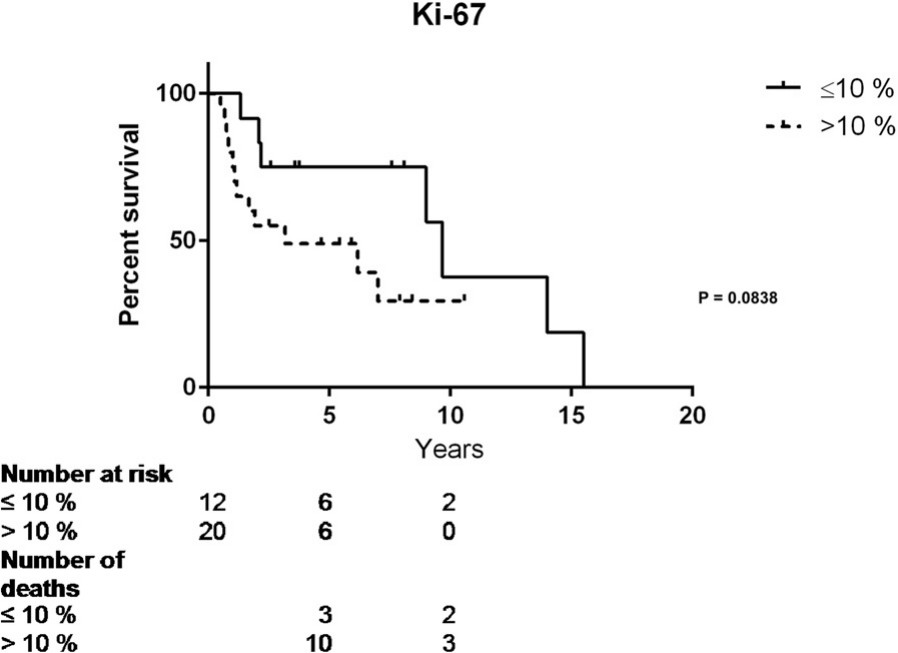
Ki-67 in 32 patients with ACC at initial surgery and survival with <10 (*n* = 12) vs. > 10 % (*n* = 20)

Patients with a normal USP had a survival time of 6.6 years (0.8–19.8) while those with a pathologic USP had a survival time of 3.3 years (0.3–19.3), however this was not statistically significant (Fig. 4). Survival did not differ significantly when analysing the proliferation with Ki-67, with a cut of-off comparisons ≤10 vs. >10 % (9.7 years [1.3–15.5] vs. 3.2 years [0.5–10.6], *P* = 0.0838) (Fig. 5).

Time to recurrence in the 25 patients in stage II with unknown metastatic disease at initial surgery was 1.5 years (0.2–9.0). At last follow-up 17 (34 %) patients were still alive. Of these nine (53 %) had been treated with cytotoxic agents whereas eight (47 %) been treated with adrenalectomy only. In patients who sought medical care with malignant symptoms 5/7 (71 %) were survivors at last follow-up, and of those with predominantly endocrine symptoms 5/19 (26 %) were still alive, however this was not statistically significant (*P* = 0.069). Eight patients (16 %) survived 10 years or more.

## Discussion

In this cohort of patients with ACC diagnosed during three decades we found a generally poor prognosis, but some were still alive without any residual disease after 19 years. Those who achieved better prognosis had been treated with radical R0 adrenalectomy, and R1 resection was also superior to R2. A normal USP compared to a pathologic pattern indicated a prolonged survival period greater than four years but this was not significant, possibly due to the limited number of ACC patients. There was also a tendency for patients without initial endocrine symptoms to have a more favourable prognosis.

The patients’ median age of 59 years is in accordance with the usual finding that the presentation age of ACC is in the fifth to sixth decade of life [27]. We also found hormonal dysfunction in 82 %, or in 93 % if urine steroid measurements were added, which is double the frequency found by others [8], but is similar to a recent review [28]. Our patients with ACC had a 5-year survival of 40 %. This is slightly more favourable than what has been reported in most previous and recent ACC series [8, 9, 29], but in line with the publication by Tran et al. [10]. ENSAT stage was clearly associated with prognosis in the present cohort, corroborating previous investigations [8, 9, 30].

The Ki-67 value ≤10 % had an implication for survival together with macroscopically radical adrenalectomy. However, in this group of patients followed for a long time the survival in patients with a Ki-67 > 10 % was not statistically different from those with lower proliferating tumours. Some of the patients with clearly elevated Ki-67 had a rapid progression of the disease as 10/20 died within 5 years (Fig. 5). Of those surviving longer the impact of Ki-67 was apparently less. The most appropriate Ki-67 cut-off level needs further investigation as Morimoto et al. found 7 % to best predict disease-free survival [19], while McNicol et al. used >3 % and related this to prolonged disease-free survival [31]. However, the latter study could not relate this to improved overall survival; probably due to lack of power as only 14 ACC patients were included. Higher cut-off levels has also been applied as <20, 20–50, and >50 % to estimate prognosis and Ki-67 was found to be the best marker for overall survival, superior to mitotic count and the mitosis specific antibody phospho-histone H3 [32]. Beuschlein et al. recently reported on a large multi-centre cohort of patients with ACC where Ki-67 was the best prognostic maker for overall survival when dividing the cohort into <10, 10–19 and ≥20 % [17]. Our findings are thus different from Beuschlein et al., which could be explained by differences in cohorts, but more probably is due to the limited number of ACC in our study. In the future tumour grade [11], and other prognostic markers may be of value in the prognostic appraisal such as gene profiling [14], steroidogenic factor 1 (SF-1) or β-Catenin [33].

A recent review stated that there was an area of uncertainty as to whether urinary gas chromatography–mass spectrometry steroid profile was of value or not [28]. We found that those with normal USP had a better prognosis compared with those with pathological USP, and those with suspected pathological USP had a prognosis in between. However, these findings were not significant possibly due to the limited number of cases and USP should be investigated in larger multi-centre cohorts of ACC. USP could also potentially be used as a tool to discover recurrence.

Interestingly we found two patients also had neurofibromatosis. Previously only nine patients with ACC have been found to also have neurofibromatosis in the literature and recently a novel germline frame shift mutation (c.5452_5453delAT) in exon 37 of the NF1 gene was described in one such patient [34].

We do not believe any benign adenoma with a normal USP may have been misdiagnosed as ACC as histopathology and other examinations clearly indicated ACC. It will be interesting to see if the recently introduced urine steroid metabolomics analysis, reported to give a sensitivity and specificity of 88 % for discriminating benign adenoma from ACC [35], will be able to predict survival in ACC.

Surprisingly, age did not have a great impact on survival. To some extent this could be explained by the fact that only 16 % of our participants were <40 years of age.

Adrenalectomy is the only possible cure in ACC [28]. However, the use of adjuvant therapy has once again increased in popularity [1], even though the evidence is still not conclusive. In our cohort 54 % had received adjuvant therapy, all with mitotane, some with streptozotocine, but it is unclear if they had a better prognosis. Of the 17 surviving patients at last follow-up, nine had been treated with adjuvant chemotherapy, whereas eight had adrenalectomy only. Thus adrenalectomy plus adjuvant chemotherapy did not seem superior to adrenalectomy alone. However, there may be a bias as it could be suspected that patients with more advanced disease more often received mitotane and/or other cytotoxic agents, but this could not be decided due to the limited number of participants.

This was a retrospective investigation with its limitations. The main limitation is the number of cases making it difficult to achieve significant results. However, since ACC is a rare disorder very few centres will have large numbers, thus multicenter and multinational studies are usually necessary to increase the number of ACC cases. Moreover, during these three decades the diagnostic and therapeutic methods changed which may have influenced the results. Some data were missing in a few patients concerning initial biochemical screening, in some cases due to the fact that the tumour type was not known at the time of surgery. The limited number of participants made subgroup analysis dependent on the year of diagnosis, meaningless. Also we could not assess Weiss score in a sufficient number of patients. A strength of the study is that all patients were well characterized and all could be followed-up.

## Conclusions

ACC has diverse clinical outcomes. Prognosis is generally poor, but some patients remain alive even many years after primary surgery, and some of these have no sign of residual disease. Radical surgery in patients without metastases in tumours with a proliferation ≤ 10 % was favourable. Urinary steroid profile has a limited prognostic value, but can give further information on endocrine disruption not displayed by traditional biochemistry.

### Ethics

This investigation complies with the current laws in Sweden. The Regional Ethical Review Board in Stockholm, Sweden approved the study.

### Availability of data

All data supporting the findings of this publication are presented in the main paper.
